# The Prevalence of Temporomandibular Disorder in Iran: A Literature Review

**DOI:** 10.30476/dentjods.2024.101406.2297

**Published:** 2025-03-01

**Authors:** Sajad Ghorbanizadeh, Kamran Azadbakht, Hamid Badrian, Nakisa Torabinia

**Affiliations:** 1 Dept. of Oral and Maxillofacial Radiology, School of Dentistry, Lorestan University of Medical Science, Khorramabad, Iran; 2 Dept. of Prosthodontics, School of Dentistry, Lorestan University of Medical Science, Khorramabad, Iran; 3 Dept. of Operative Dentistry, School of Dentistry, Lorestan University of Medical Science, Khorramabad, Iran; 4 Dept. of Oral and Maxillofacial Pathology, Dental Materials Research Center, Dental Research Institute, School of Dentistry, Isfahan University of Medical Sciences, Isfahan, Iran

**Keywords:** Temporomandibular Joint disorder, Prevalence, Iran

## Abstract

**Statement of the Problem:**

Temporomandibular joint disorder (TMD) will appear if there is a problem with the temporomandibular joint, bones, related muscles, or ligaments. This complication causes severe joint pain near the ears, head, neck, and jaws. TMD has been reported to affect 40 to 70% of adults.

**Purpose:**

Based on previous studies, the present review aimed to determine the prevalence of TMD among the Iranian population.

**Materials and Method:**

This review and meta-analysis was performed according to the PRISMA guidelines. All relevant studies published during 2000-2023 were retrieved by a systematic search in available international databases, including Web of Science, Science Direct, Scopus, PubMed, and Google Scholar, and domestic Persian databases, including SID, Magiran, and Iran Medex. Finally, 22 completely related studies were selected to investigate the main objective. The Comprehensive Meta-analysis (CMA) software was used for data analysis in this systematic review.

**Results:**

Initially, 212 articles were retrieved, of which 116 were duplicate studies. Further, 39 studies were excluded after evaluation of the title and abstract, and 35 studies were excluded after considering the inclusion and exclusion criteria. Finally, 22 articles were included in the meta-analysis. The pooled prevalence of TMD in Iran was 0.56 (0.44-0.68).

**Conclusion:**

In general, the prevalence of TMD in the Iranian population is relatively high. Therefore, it is necessary to develop strategies to educate people, especially those at risk. Furthermore, due to the presence of TMD in children and students in some parts of the country, it is necessary to perform essential examinations in preschools to prevent the development of this disorder in later life.

## Introduction

Temporomandibular joint (TMJ) is important for chewing, swallowing, speaking, and even breathing. In addition, TMJ is the only joint in the body that is made of the connection between two symmetrical joints that act in harmony [ 1 ]. Therefore, temporomandibular disorder (TMD) is a term that refers to problems with the masticatory system, which includes the TMJ, the musculoskeletal system, and the supporting bone. TMD has been reported to affect 40 to 70% of adults, while its prevalence in children with deciduous teeth and a mixed dental system reaches 16% and 90% [ 2 - 5 ].

TMD is a multifactorial disease with acquired and hereditary factors. The acquired factors include infection, injuries, surgery, radiation therapy, habits, tumors, etc. The hereditary factors consist of hemifacial microsomia, hemifacial atrophy, juvenile rheumatoid arthritis, oncology, muscle spasm, ectopic occlusal contact, stress, systemic disease, and immunological factors [ 5 ]. 

To diagnose the signs and symptoms of TMD, a complete medical and dental history is first obtained from the patient to discover any hereditary or acquired disorder. In addition, a history of trauma and pain should be provided. Then, the patient’s clinical examination begins with palpation of the posterior temporal, medial, and anterior muscles, a superficial and deep masseter, lateral pterygoid, sternocleidomastoid, superior trapezius, suboccipital and posterior neck muscles [ 5 - 7 ]. Then, several parameters are essential to be considered, which include touching the TMJ in the opening and closing position of the mouth, lateral movements, pain in touching, the presence of joint sounds, initial deviation in opening the mouth, jaw returning to the middle position (deviation), continuous deviation of the jaw to the end deflection, the patient’s degree of prevention, and occlusion [ 5 ].

The most common signs and symptoms of TMD are hamstring and TMJ pain, muscle dysfunction, joint sounds, headache, abnormal jaw movement, mouth opening, abrasions, and tingling [ 3 , 5 , 8 - 9 ]. It should be noted that the incidence of TMD symptoms increases with age increase [ 8 ]. Children, however, have difficulties describing pain and identifying its source, and the relationship between signs and symptoms is not clear in children [ 5 ].

Since TMD is the most common jaw disorder that affects the masticatory muscles, bony components of the TMJ, and soft tissue fragments of TMJ (especially the articular disc and ligament joints), it is important to survey this complication in society. In addition, no review study has been conducted on the prevalence of TMD in Iran, so it is necessary to conduct a systematic review to shed more light on this lacuna.

## Materials and Method

### Searched databases and search strategy

This study aimed to investigate the prevalence of TMD among the Iranian population. For this purpose, systematic searches of internationally available databases, including Web of Science, Science Direct, Scopus, PubMed, and Google Scholar, were performed between 2000 and 2021. In addition, databases with the Persian language, such as SID, Magiran, and Iran Medex, included Farsi keywords. Systematic reviews were performed using Mesh terms "Temporomandibular", "Joint", "Disorders", "Iran", "TMD", "TMJ", "Dental" and "Prevalence", "Occlusion", "Signs", "Symptoms ","Patients" and "Dental". For other databases, the same Mesh terms were used similarly. In addition, unofficial reports, articles in a letter-to-editor format, and unpublished articles and content posted on internet sites were removed from the list of downloaded files.

### Inclusion and exclusion criteria

The inclusion criteria were studies whose abstracts and full texts were available, and studies not reporting the desired statistical parameters were excluded.

### Study selection and data collection

**Figure 1:**
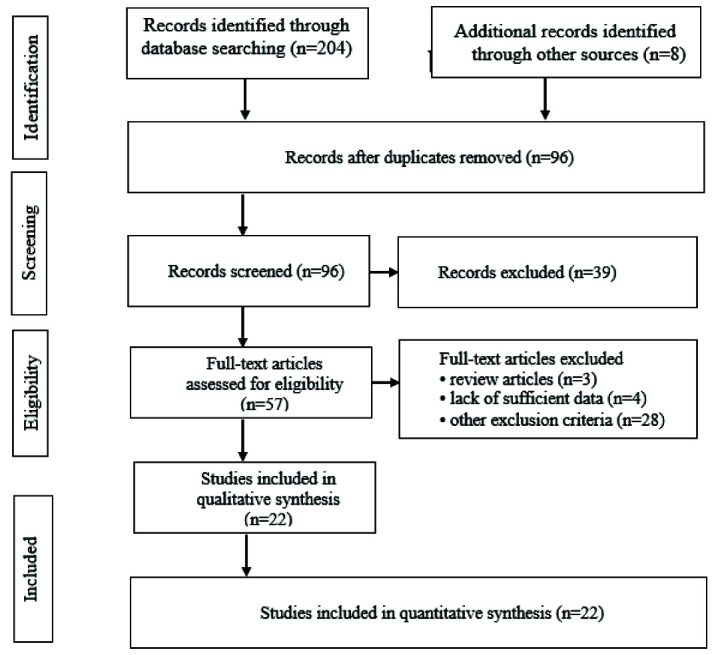
Flow diagram of study identification according to PRISMA

After the electronic search of all databases, screening was done in three phases by the authors separately, including phases I, II, and III, to determine the eligibility of studies. In phase I, the titles and abstracts of articles were checked, and in phase II, studies with unrelated titles or not matching the included criteria were deleted. In phase III, the final selected full-text articles were evaluated to extract the desired results. Furthermore, the references of full-text articles were thoroughly evaluated to verify that no articles were missed for inclusion in the study (reference checking). In addition, the citations from the full-text articles were checked (citation tracing) to make sure that the search was thorough and successful. The two researchers independently examined the quality and risk of bias of studies, and the disagreements between them were resolved through discussion or consultation with a third researcher. Finally, 22 published articles were reviewed in the present study (Figure 1). The literature search for articles was done according to the PRISMA guidelines [ 10 ]. 

### Data analysis

The Comprehensive Meta-analysis (CMA) software was used to analyze the data. After feeding the information of the articles into the CMA, the I^2^ and Qvalue tests were used to detect the heterogeneity of the studies. If the I^2^ index was high, the random model was used; otherwise, the fixed model was employed. Begg’s and Egger’s tests as well as the funnel plot were used to evaluate the dispersion bias. 

## Results

### Study selection

In the initial search of international and domestic data bases until April 2021, 204 articles were retrieved. The manual search also yielded 8 articles. Of these, 116 articles were duplicates and excluded. After reviewing the title and abstract of the remaining 96 articles, 39 irrelevant articles were excluded. After the fulltext analysis of the remaining 57 articles, 35 articles were excluded. 

### Descriptive results

In this review, the results of 22 studies were extracted, which can be seen in Table 1. These results included the study year, research population, study location, age category, gender, sample size, and prevalence of TMD. In addition, other key results for each study are presented in Table 1.

**Table 1:** The results of previous studies on the prevalence of temporomandibular disorder (TMD) in Iran

Authors (study year)	Research population	Study location	Age category (year)	Gender (sample, size, person)	Prevalence of TMD (%)	Other key results
Baradaran Nakhjavani *et al.* (2012) [ 11 ]	Elementary school students	Tehran	Range: 7-9	Female (205) and male (195)	65.2	Class III occlusion, bruxism, and Deep Bite
Mahshid *et al.* (2007) [ 33 ]	Ordinary people	Tehran	Range: 18-77	Female (839) and male (64)	85.7	Bruxism and trauma
Jahandideh *et al.* (2017) [ 16 ]	Patients referred to dentistry centers	Rasht	Range: 7-9	Female (282) and male (218)	62.6	
Gavahi *et al.* (2019) [ 34 ]	Patients with mandibular fracture	Yazd	Range: 3-59	Female (70) and male (225)	95	
			7-9			
Mohajerani *et al.* (2018) [ 17 ]	Patients referred to dentistry centers	Tehran	<27, 27-45, >45 (193) and male (141	Female Three groups	91	
Anbari *et al.* (2020) [ 35 ]	Law students	Bandar-e- Anzali	Range: 18-25	Female (67) and male (33)	43	Hidden anxiety
Falahati *et al.* (2020) [ 19 ]	Patients referred to dentistry centers	Isfahan	Mean: 33.8	Female (101) and male (101)	43.6	Orthodontic treatment (*p*= 0.42) and parafunctional habits (*p*= 0.46) were not significantly different between the two groups (with and without TMD)
Bahrani *et al.* (2012) [ 36 ]	Dental and non-dental students	Shiraz	Range: 18-30	Female (100) and male (100)	Dental students: 80	
					Non-dental students: 62	
Ebrahimi *et al.* (2011) [ 14 ]	High school students	Mashhad	Range: 14-18	Female (400) and male (40	34.7	Clicking, muscle tenderness and temporomandibular joint (TMJ) tenderness
Banki *et al.* (2023) [ 21 ]	dental student	Golestan	Range: 18-28	98 male-95 females	80.8	Bruxism and trauma history
Nokar *et al.* (2019) [ 39 ]	Patients with TMD	Tehran	Range: 15-65	Female (65) and male (58)	69	Occlusal factors playing a role in the etiology of TMD
			Mean: 36.6			
Mirmohamadsadeghi *et al.* (2019) [ 39 ]	Patients with and without TMD chosen before the third molar surgery	Tehran	Range: 15-30	Total female and male (71)	39	-
			Mean: 24.3			
Fariaby and Mohammad (2005) [ 12 ]	Elementary school students	Kerman	Range: 9-12	Male (240)	11.7	Limitations in mouth opening, deviation in jaw opening
Ebrahimi Saravi *et al.* (2016) [ 20 ]	Patients referred to dentistry center	Sari	NR	Female (41) and male (27)	75	Among 68 patients, muscle pain
Jahanimoghadam *et al.* (2023) [ 40 ]	Elementary school students	Kerman	Mean: 9.18	327 male-273 female	16.5 female- 16.9 male	Awake and sleep bruxism
Baghaee *et al.* (2008) [ 13 ]	Preschool children	Mashhad	6	Female (221) and male (231)	44.2	Clicking, crepitus, deviation
Balke *et al.* (2010) [ 41 ]	Attendees of medical healthcare centers	Mashhad and Zoshk	Urban: [range: 18-64, mean: 33.68± 10.31]	Female (171) and male (52)	Urban: 24.4	Disc displacement
			Rural: [range: 18-65, mean: 32.07± 10.83]		Rural: 31.7	
Hashemipour *et al.* (2018) [ 15 ]	First- to-fourth-grade high school students	Kerman	Range: 14-18, Mean: 15.0±1.1	Total female and male (368)	79	Pain in masticatory muscles, pain at mouth opening

### Analytical results

**Figure 2:**
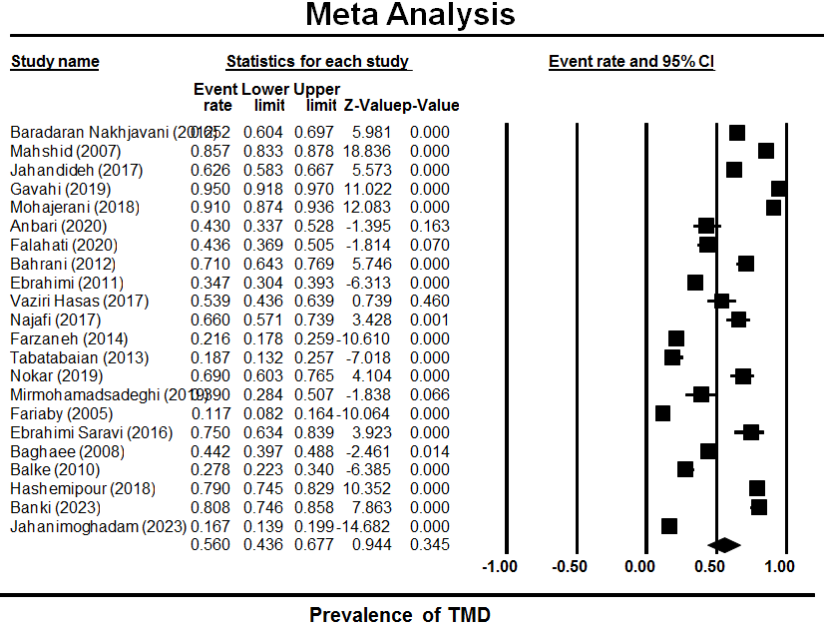
The results of previous studies on the prevalence of temporomandibular disorder (TMD) in Iran

**Figure 3:**
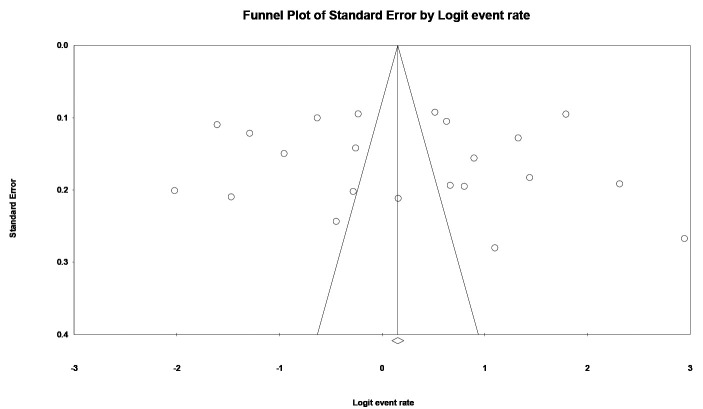
Funnel plot of standard error

The meta-analysis done on previous studies showed that the pooled prevalence of TMD in Iran is 0.56 (0.44-0.68) (Figure 2). According to the funnel plot, the distribution of published studies was symmetric, and also the Begg’s test indicated no bias in the study (*p* Value= 0.37) (Figure 3).

## Discussion

The present metaanalysis investigated the prevalence of TMD in Iran. Most of the studies included in this meta-analysis showed that the prevalence of TMD in the Iranians is relatively high (56%). According to different studies, the prevalence of TMD has been investigated in students. The results of studies done by Baradaran Nakhjavani and Fardi (2012) in Tehran, Fariaby and Mohammadi (2005) in Kerman, and Baghaee *et al.* (2008) [ 11 - 13 ] in Mashhad showed the prevalence of TMD among elementary school students in the age range of 7-12 years was 11.7%-65.2%. This value, however, was 79% among high school students in the studies done by Ebrahimi *et al.* in Mashhad (2011) and Hashemipour *et al.* in Kerman (2018) [ 14 - 15 ]. Some studies have also focused on the prevalence of TMD in patients referred to dental centers. In the study of Jahandideh in Rasht (2017), the prevalence of TMD was 66.6% [ 16 ], while it was reported to be 91% by Mohajerani (2018) [ 17 ] and 14.4% by Sahebi and Bostani (2010) in Tehran [ 18 ]. The prevalence of TMD has also been reported to be 43.6% by Falahati in Isfahan (2020) [ 19 ] and 91% by Ebrahimi Saravi (2016) in Sari [ 20 ]. 

According to these results, it can be argued that TMD disorders are the main reason for the referral of more than 40% of adults to dental clinics. The variation in the results of various studies could be related to differences in sample size, the multifactorial nature of this problem, the role of different etiologic factors, and variable diagnostic methods for the evaluation of TMD.

The most common reported symptoms of TMD were unilateral or bilateral articular sound, jaw deviation, limitation of mouth opening, muscle tenderness, and pain in the maxillary joint [ 21 - 28 ].

In most similar studies, the prevalence of TMD was higher in women than in men. For example, in the studies of Kitsoulis *et al.* (2011) [ 29 ], Shetty *et al.* (2010) [ 30 ], Mohajerani *et al.* (2018) [ 17 ], In addition, Lasemi Saravi *et al.* [ 31 ] (2008) found that gender is an influential factor in TMD frequency. 

According to the studies of Jahandideh *et al.* (2017), Lasemi Saravi *et al.* (2008), and Johansson *et al.* (2003), the prevalence of TMD was significantly higher in people with parafunctional habit than in those without this habit and also in people with a history of trauma than in people without a history of trauma [ 16 , 31 - 32 ]. Thus, parafunctional habits and trauma can be considered the risk factors for TMD. 

Based on the results of the previous studies, the prevalence of TMD is relatively high among Iranian patients referred to dental centers. Therefore, planning the necessary education for people in the community, especially the high-risk individuals (older age group, people with oral parafunctional habits, and history of trauma, jaw dislocation, and loss of posterior teeth), can effectively prevent these TMJ complications. In addition, increasing dentists’ awareness of the clinical symptoms of this disorder can help differentiate TMD pain from a variety of headaches, earaches, and other neurological pains and help treat the patients.

### Limitation

There was a problem accessing some databases and full-text article.

## Conclusion

In general, the pooled prevalence of TMD in Iranians is 0.56 (0.44-0.68). Oral parafunctional habits and trauma can affect the incidence of TMD. The most common symptoms of TMD include joint sounds, jaw deviation when opening the mouth, muscle tenderness, and masticatory muscle pain. 

## References

[1] Scrivani SJ, Keith DA, Kaban LB ( 2008). Temporomandibular disorders. N Engl J Med.

[2] Okeson JP ( 2019). Management of temporomandibular disorders and occlusion-E-book.

[3] Castelo PM, Gavião MB, Pereira LJ, Bonjardim LR ( 2005). Relationship between oral parafunctional/nutritive sucking habits and temporomandibular joint dysfunction in primary dentition. Int J Paediatr Dent.

[4] Mackie A, Lyons K ( 2008). The role of occlusion in temporomandibular disorders--a review of the literature. N Z Dent J.

[5] Pinkham J, Casamassimo P, Fields HW ( 2005). Pediatric dentistry: Infancy through adolescence.

[6] Conti AC, Oltramari PV, Navarro RD, Almeida MR ( 2007). Examination of temporomandibular disorders in the orthodontic patient: a clinical guide. J Appl Oral Sci.

[7] Bonjardim LR, Gavião MB, Pereira LJ, Castelo PM ( 2004). Mandibular movements in children with and without signs and symptoms of temporomandibular disorders. J Appl Oral Sci.

[8] de Souza Barbosa T, Miyakoda LS, de Liz Pocztaruk R, Rocha CP, Gavião MB ( 2008). Temporomandibular disorders and bruxism in childhood and adolescence: review of the literature. Int J Pediatr Otorhinolaryngol.

[9] Cooper BC, Kleinberg I ( 2007). Examination of a large patient population for the presence of symptoms and signs of temporomandibular disorders. CRANIO®.

[10] Moher D, Liberati A, Tetzlaff J, Altman DG ( 2010). Preferred reporting items for systematic reviews and meta-analyses: the PRISMA statement. Int J Surg.

[11] Baradaran Nakhjavani Y, Fardi M ( 2012). Prevalence of temporomandibular disorders in children age 7-9 years in primary schools of tehran. J Res Dent Sci.

[12] Fariaby J, Mohammadi M ( 2005). Prevalence of temporomandibular joint disorders in 9-12-year-old boy students in Kerman, Southeast of Iran. Iran J Med Sci.

[13] Baghaee B, Ajami B, Hafez B, Khaleseh N, Sarraf Shirazi A ( 2009). Evaluation of the relationship between occlusion and temporomandibular disorders in six-year-old preschool children in Mashhad-Iran. J Mashhad Dent.

[14] Ebrahimi M, Dashti H, Mehrabkhani M, Arghavani M, Daneshvar-Mozafari A ( 2011). Temporomandibular disorders and related factors in a group of Iranian adolescents: a cross-sectional survey. J Dent Res Dent Clin Dent Prospects.

[15] Hashemipour MA, Moslemi F, Mirzadeh A, Mirzadeh A ( 2018). Parafunctional habits and their relationship with temporomandibular joint disorders in Iranian school students. Meandros Med Dental J.

[16] Jahandideh Y, Basirat M, Tayefeh Davalloo R ( 2017). Prevalence of temporomandibular disorders and the associated factors. J Guilan Univ Med Sci.

[17] Mohajerani SH, Baghnoee AM, Ghorbani Z, Gholami L, Tavakolizadeh S, Ebrahimzadeh Z ( 2018). Prevalence of temporomandibular disorders among patients referred to Shahid Beheshti Dental School, Iran (2007-2008). Avicenna J Dent Res.

[18] Sahebi M, Bostani AP ( 2010). Prevalence of temporomandibular disorders and its association with malocclusion in mixed dentition among patients referred to Tehran University Dental School. Int J Dent Med.

[19] Falahati M, Golmohammadi F, Darabi R, Jafari M ( 2020). Evaluation of temporomandibular joint disorders and related factors in patients referring to dental school of Isfahan Islamic Azad University in 2019. J Res Dent Maxillofac Sci.

[20] Ebrahimi Saravia M, Khalilian A, Ronaghi H ( 2016). Prevalence of temporomandibular disorders (TMD) and its signs and symptoms in sari dental school clinic. J Maz Univ Med Sci.

[21] Banki M, Javaherian A, Tahmasebi P, Farsinia F, Sarbazdalir A, Eslami N, et al ( 2023). Prevalence of signs and symptoms of temporomandibular joint disorders and associated factors among Iranian dental student. World J Biolog Pharm Health Sci.

[22] Najafi S, Tafakhori A, Fard MJ, Radfar L ( 2017). Prevalence of temporomandibular disorder in patients with chronic headache. J Dentomaxillofac Res.

[23] Farzaneh B, Salari S, Fekrazad R ( 2014). Prevalence of temporomandibular joint disorder and stress related dental attritions among army personnel. J Arch Mil Med.

[24] Gouharian R, Madani AA ( 2006). Evaluation of temporomandibular joint status and related signs and symptoms in students of Mashhad Dental School. Iran. J Otorhinolaryngol.

[25] Kritsineli M, Shim YS ( 1992). Malocclusion, body Posture and TMD in children with mixed and permanent dentition. J Clin Pediat Dent.

[26] Kamisaka M, Yatani H, Kuboki T, Matsuka Y, Minakuchi H ( 2000). Four-year longitudinal course of TMD symptoms in an adult population and the estimation of risk factors in relation to symptoms. J Orofac Pain.

[27] Bonjardim LR, Gaviao MB, Cormagnani FG, Pereira LJ, Castolo P ( 2003). Sign and symptom of TMD in children with primary dentition. J Clin Pediat Dent.

[28] Gesch D, Bernhardt O, Alte D, Schwahn C, Kocher T, John U, et al ( 2004). Prevalence of signs and symptoms of temporomandibular disorders in an urban and rural German population: results of a population-based Study of Health in Pomerania. Quintessence Int.

[29] Kitsoulis P, Marini A, Iliou K, Galani V, Zimpis A, Kanavaros P, et al ( 2011). Signs and symptoms of temporomandibular joint disorders related to the degree of mouth opening and hearing loss. BMC Ear Nose Throat Disord.

[30] Shetty R ( 2010). Prevalence of signs of temporomandibular joint dysfunction in asymptomatic edentulous subjects: A cross-sectional study. J Indian Prosthodont Soc.

[31] Lasemi E, Navi F, Basir Shabastari S ( 2008). Prevalence of temporomandibular disorders and it’s related factors in dental school of Azad University of Tehran in 2005. J Mashhad Dent.

[32] Johansson A, Unell L, Carlsson GE, Söderfeldt B, Halling A ( 2003). Gender difference in symptoms related to temporomandibular disorders in a population of 50-year-old subjects. J Orofac Pain.

[33] Mahshid M, Ajlali M, Nori M, Droodian AA, Shalchizadeh A ( 2007). Prevalence of temporomandibular disorders in Tehran Health Centers in summer 2002. J Dent Sch.

[34] Gavahi M, Abbaszadeh F ( 2019). Relative frequency of temporomandibular joint disorder in the patients with mandibular fracture in Yazd city during 2015-2017. J Shahid Sadoughi Univ Med Sci.

[35] Anbari F, Yazdani Kachooei Z, Salemi M, Anbari F ( 2020). Anxiety and temporomandibular joint disorders among law students in Iran. J Dentomaxillofac Res.

[36] Bahrani F, Ghadiri P, Vojdani M ( 2012). Comparison of temporomandibular disorders in Iranian dental and nondental students. J Contemp Dent Pract.

[37] Tabatabaian F, Saboury A, Ghane HK ( 2013). The prevalence of temporomandibular disorders in patients referred to the prosthodontics department of Shahid Beheshti Dental School in fall 2010. J Dent Sch.

[38] Nokar S, Sadighpour L, Shirzad H, Shahrokhi Rad A, Keshvad A ( 2019). Evaluation of signs, symptoms and occlusal factors among patients with temporomandibular disorders according to Helkimo index. CRANIO®.

[39] Mirmohamadsadeghi H, Alavi O, Karamshahi M, Tabrizi R ( 2019). Prevalence of temporomandibular joint problems in candidate patients for impacted third molar surgery with and without the previous temporomandibular disorder: a prospective study. Dent Hypotheses.

[40] Jahanimoghadam F, Tohidimoghadam M, Poureslami H, Sharifi M ( 2023). Prevalence and risk factors of bruxism in a se-lected population of Iranian children. Pesqui Bras Odon-topediatria Clín Integr.

[41] Balke Z, Rammelsberg P, Leckel M, Schmitter M ( 2010). Prevalence of temporomandibular disorders: samples taken from attendees of medical healthcare centers in the Islamic Republic of Iran. J Orofac Pain.

